# Monitoring and Prognostic Analysis of Severe Cerebrovascular Diseases Based on Multi-Scale Dynamic Brain Imaging

**DOI:** 10.3389/fnins.2021.684469

**Published:** 2021-06-30

**Authors:** Suting Zhong, Kai Sun, Xiaobing Zuo, Aihong Chen

**Affiliations:** ^1^Department of Emergency Medicine, Hanyang Hospital, Wuhan University of Science and Technology, Wuhan, China; ^2^Department of Neurosurgery, Yantai Penglai Traditional Chinese Medicine Hospital, Yantai, China

**Keywords:** severe cerebrovascular disease, dynamic brain imaging, multi-scale features, ApacheII score, monitoring and prognostic analysis

## Abstract

Severe cerebrovascular disease is an acute cerebrovascular event that causes severe neurological damage in patients, and is often accompanied by severe dysfunction of multiple systems such as breathing and circulation. Patients with severe cerebrovascular disease are in critical condition, have many complications, and are prone to deterioration of neurological function. Therefore, they need closer monitoring and treatment. The treatment strategy in the acute phase directly determines the prognosis of the patient. The case of this article selected 90 patients with severe cerebrovascular disease who were hospitalized in four wards of the Department of Neurology and the Department of Critical Care Medicine in a university hospital. The included cases were in accordance with the guidelines for the prevention and treatment of cerebrovascular diseases. Patients with cerebral infarction are given routine treatments such as improving cerebral circulation, protecting nutrient brain cells, dehydration, and anti-platelet; patients with cerebral hemorrhage are treated within the corresponding safe time window. We use Statistical Product and Service Solutions (SPSS) Statistics21 software to perform statistical analysis on the results. Based on the study of the feature extraction process of convolutional neural network, according to the hierarchical principle of convolutional neural network, a backbone neural network MF (Multi-Features)—Dense Net that can realize the fusion, and extraction of multi-scale features is designed. The network combines the characteristics of densely connected network and feature pyramid network structure, and combines strong feature extraction ability, high robustness and relatively small parameter amount. An end-to-end monitoring algorithm for severe cerebrovascular diseases based on MF-Dense Net is proposed. In the experiment, the algorithm showed high monitoring accuracy, and at the same time reached the speed of real-time monitoring on the experimental platform. An improved spatial pyramid pooling structure is designed to strengthen the network’s ability to merge and extract local features at the same level and at multiple scales, which can further improve the accuracy of algorithm monitoring by paying a small amount of additional computational cost. At the same time, a method is designed to strengthen the use of low-level features by improving the network structure, which improves the algorithm’s monitoring performance on small-scale severe cerebrovascular diseases. For patients with severe cerebrovascular disease in general, APACHEII1, APACHEII2, APACHEII3 and the trend of APACHEII score change are divided into high-risk group and low-risk group. The overall severe cerebrovascular disease, severe cerebral hemorrhage and severe cerebral infarction are analyzed, respectively. The differences are statistically significant.

## Introduction

Severe cerebrovascular disease is a critical type of acute cerebrovascular disease, which causes severe neurological damage in patients, and is often accompanied by severe dysfunction of multiple systems such as breathing and circulation (Conzen et al., 2019). Because patients with severe cerebrovascular disease are critically ill, have many complications, and are prone to deterioration of neurological function, they often need to be transferred to the intensive care unit for closer monitoring and treatment (Zafar et al., 2019; Xu et al., 2020). The activation of the neuroendocrine system, the increase in intracranial pressure, and a history of previous hypertension after acute stroke often lead to increased blood pressure (Václavů et al., 2019). Therefore, blood pressure management is one of the basic links and one of the crucial links in the acute treatment of patients with severe cerebrovascular disease (Rosenberg et al., 2020). Elevated blood pressure can lead to encephalopathy, cardiac complications, and kidney damage. In patients with spontaneous cerebral hemorrhage, it can also cause hematoma enlargement, aggravate the edema of the tissue around the hematoma, and cause rebleeding (Savić et al., 2019). However, excessive reduction in blood pressure can increase the degree of cerebral ischemia in patients with acute ischemic stroke by reducing cerebral perfusion pressure, and will also significantly increase the risk of hematoma enlargement, rebleeding, death, and disability in patients with spontaneous cerebral hemorrhage (Rocha and Singhal, 2020).

In the neurological intensive care unit, the second injury in the course of most patients with acute brain injury will significantly increase the rate of deterioration and mortality (Robertson et al., 2019). Early detection and early prevention of these complications are necessary. Before the general neurological examination is positive, the brain function or structure has undergone irreversible changes. The brain function monitoring will provide effective information during the reversible period of neurological dysfunction, early detection of the second injury, help clinicians to intervene in time to prevent continuous brain damage, and continuous evaluation of the treatment effect (Mathieu et al., 2020). EEG (Electroencephalogram) is more sensitive to abnormal brain metabolism, ischemia, hypoxia and abnormal nerve function, and can be used as one of the methods to monitor brain function in the NICU (Neonatal Intensive Care Unit) (Merikangas et al., 2019). It can determine non-convulsive seizures (NCS), non-convulsive status epilepticus (NCSE), guide the drug treatment of epilepsy, predict and detect cerebral ischemia, monitor cerebral edema and intracranial pressure, and help predict the outcome of coma patients (Quon et al., 2019). EEG is closely related to the metabolism of brain nerve cells. It is sensitive to brain damage caused by ischemia and hypoxia. Nervous system dysfunction can be found in the reversible phase of brain damage, and the recovery of nerve function can be found when other clinical examinations have failed (Rahmim et al., 2019). It is the most effective means to discover epileptic activity. In addition, CEEG (Clinical Electroencephalogram) monitoring can provide dynamic information about brain function, which is conducive to real-time monitoring of brain function changes. The rapid development of computer and computer network technology makes the implementation and wide application of CEEG monitoring possible (Tanaka et al., 2020). Digital EEG (DEEG) has become the preferred technology of CEEG. It makes it easy to store a large amount of EEG information, which can be edited, quantitatively analyzed, automatically removed artifacts, and automatically identified abnormal waveforms (Marini et al., 2020). CEEG accurately arranges disk electrodes in accordance with international standards. Monitoring requires good scalp electrode fixation. Generally, electrode paste is adhered to the scalp surface and fixed with collodion, which is feasible for long-term monitoring (Lohith et al., 2019; Malas et al., 2019). The standard EEG recorder records CEEG in the storage medium in digital format. In NICU, DEEG sampling frequency of 128–256/channel/s can provide enough information. The detailed EEG data of each patient every day can be stored up to 2GB. After obtaining the EEG data, the NICU staff needs to review and summarize, and the EEG technicians and EEG experts will observe and analyze, and draw conclusions (Taneja et al., 2019; Maïer et al., 2020). Although NICU nurses can recognize some typical EEG after training, some complicated CEEG records still require detailed explanation by EEG experts (Waddle et al., 2020). This has always been a problem that restricts the widespread use of CEEG for NICU monitoring and needs to be further studied. The original EEG is converted into QEEG (Quantified Electroencephalogram). It mainly includes power spectrum analysis, time domain analysis, formation of compressed spectrum array and EEG topographic map (Khalil et al., 2020). QEEG can quickly display important EEG changes, such as epileptic waves, slow wave, general suppression, lack of fast wave, increase and decrease of EEG variability, etc. But at present, QEEG still cannot be used in clinic separately from the original EEG (Koskinen et al., 2019).

Nerve cells are very sensitive to ischemic damage, and the pyramidal cells of the third and fifth layers of the cerebral cortex, which are closely related to the production of EEG, are most vulnerable to ischemic damage (Wang C. et al., 2019). Ischemia damage causes changes in the posterior potential of cerebral cortex neurons, and abnormal information can be recorded in the scalp EEG. When the cerebral blood flow (CBF) drops to 25–30 ml/100 g/min, the neuronal dysfunction is in a reversible phase, and the EEG changes (Wang Y. et al., 2019). It is often manifested as a lack of α and β activities, and theta waves and delta waves appear one after another. When CBF drops below 10–12 ml/100 g/min, nerve cells will be irreversibly damaged, and EEG will also be abnormal (Li et al., 2020). Therefore, EEG is very sensitive to cerebral ischemia during the reversible period of neuronal dysfunction, and there are general changes. Clinical timely intervention can prevent continuous brain damage. On the other hand, EEG is also very sensitive to the recovery of cerebral ischemia, and it is found earlier than clinical examination. Cerebral edema and increased intracranial pressure in the course of cerebral hemorrhage patients are the main reasons for the deterioration and death of the disease. Monitoring it is the key to prevention and treatment (Wardlaw et al., 2019). In patients with cerebral edema and increased intracranial pressure, EEG shows continuous diffuse wave activity, and EEG is significantly improved after using dehydrating agents such as mannitol (Zeiler et al., 2020). CEEG monitoring can not only be used to monitor and reflect dehydration of dehydrating agents in treatment of cerebral edema, but also can provide early effects of drug treatment. Compared with CT, MRI, and other examinations, CEEG has the advantage of continuous, dynamic and real-time bedside monitoring (Liu et al., 2019). Transcranial Doppler (TCD) ultrasound can evaluate the cerebral perfusion and intracranial pressure of patients with severe cerebrovascular disease by monitoring the changes of hemodynamic parameters, such as blood flow direction, blood flow velocity, spectrum shape, pulsation index, etc. After severe cerebrovascular disease occurs, intracranial pressure changes sharply, and brain herniation often occurs (Forti et al., 2019; Dumitrascu and Koronyo-Hamaoui, 2020). The initial manifestation of TCD changes when the intracranial pressure increases, the systolic peak becomes sharp, and the diastolic blood flow signal disappears when the intracranial pressure approaches the diastolic pressure, leaving only the systolic sharp wave; the intracranial pressure continues to increase, when the diastolic blood pressure is exceeded, the diastolic blood flow reappears, but the direction is negative, which is a “shock wave,” indicates that the compensation mechanism of cerebrovascular is not enough to counter the increase in intracranial pressure. It shows needle-like blood flow in the early stage of contraction, that is, “nail wave.” At this time, it is difficult for peripheral blood flow to enter the cerebral circulation, which is a characteristic manifestation of cerebral circulation stop (Dehkharghani and Qiu, 2020). The flow is getting smaller and smaller, and eventually the blood flow stops.

In this experimental study, the EEG patterns of severe brain patients were also analyzed and studied, and it was pointed out that the prognosis of patients with a-wave coma was relatively poor. In severe patients without a wake-sleep cycle, if some sleep spindle components are added to the slow wave or a wave background, it indicates that the patient has a better prognosis, especially the long-term EEG monitoring in the diagnosis, treatment and evaluation of acute and severe cerebrovascular diseases. Aiming at the monitoring problem of severe cerebrovascular disease in multi-scale dynamic brain imaging, the bottleneck of the existing convolutional neural network for monitoring severe cerebrovascular disease in the multi-scale feature extraction is analyzed, and the dense connection network structure and the feature pyramid network structure are combined to design a convolutional neural network MF-Desne Net that can realize multi-scale feature fusion extraction. Based on this network, an end-to-end multi-scale dynamic brain imaging monitoring algorithm for severe cerebrovascular disease MDRD (Multi-scale Dense ResNet Detector) was designed. Experiments show that the MDRD algorithm not only achieves high monitoring accuracy, but also has a fast monitoring speed, which can meet the needs of real-time monitoring. At the same time, on the basis of the above work, the multi-scale feature extraction of convolutional neural network is further studied. A three-dimensional spatial pyramid pooling method is proposed to improve the network’s ability to fuse and extract local multi-scale features of the image; a method to strengthen the use of low-level features based on reconstruction operations is proposed, which improves the network’s ability to use low-level extraction capacity. APACHEII1, APACHEII2, and APACHEII3 are related to prognosis. The higher the score, the higher the risk of death. The APACHEII1, APACHEII2, and APACHEII3 of the death group are higher than those of the survival group. The scores of APACHEII1, APACHEII2, and APACHEII3 according to Youden index are 19, 19, and 17 points, respectively. It shows that the score of patients with severe cerebrovascular disease is higher than 19 in the first 24 h after admission to the NICU, the score is still higher than 19 in the second 24 h, and the score does not fall below 17 in the third 24 h.

The rest of this article is organized as follows. Section “Data and Methods for Monitoring Severe Cerebrovascular Disease” discusses the data and methods of monitoring severe cerebrovascular diseases. In section “Multi-Scale Dynamic Brain Imaging Monitoring Model for Severe Cerebrovascular Disease Based on Feature Fusion”, a multi-scale dynamic brain imaging monitoring model of severe cerebrovascular disease based on feature fusion is constructed. In section “Analysis of the Results of Monitoring and Prognosis of Severe Cerebrovascular Disease”, the results of the surveillance and prognosis experiments of severe cerebrovascular disease were analyzed. Section “Conclusion” summarizes the full text.

## Data and Methods for Monitoring Severe Cerebrovascular Disease

### Clinical Features of Severe Cerebrovascular Disease

In clinical practice, patients with severe cerebrovascular diseases have varying degrees of consciousness disturbance, and the prognosis is poor. Patients with severe cerebrovascular diseases are often prone to hospital-acquired pneumonia due to the age of the patient, poor patient awareness, poor cough and sputum expectoration, and the use of multiple medical equipment, which will seriously affect the prognosis of the patient. In addition, it is neurogenic. Pulmonary edema can also aggravate the progression of pneumonia. Severe cerebrovascular disease is accompanied by multiple complications, such as pneumonia, electrolyte imbalance and concurrent seizures, and it is found that patients with concurrent pulmonary infection and internal environmental disorders indicate a higher mortality rate. Half of the patients included in the study also developed pneumonia to varying degrees, and the patients developed severe pneumonia and were infected with multi-drug resistant bacteria. The patient underwent tracheotomy, confirming that pneumonia is a common complication of severe cerebrovascular disease.

**FIGURE 1:**
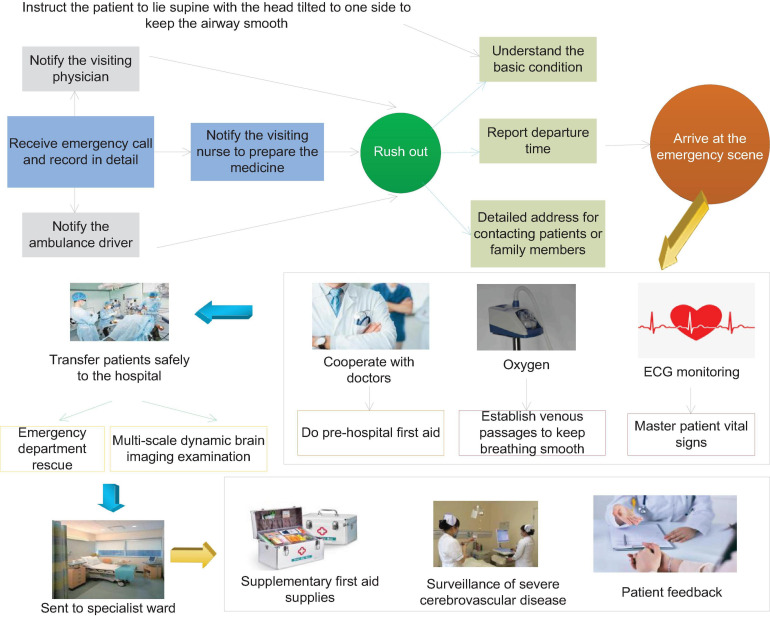
Flow chart of emergency care for patients with severe cerebrovascular disease.

More than half of the surviving patients with severe cerebrovascular disease will have different degrees of disability, such as paralysis of the lateral limbs, various types of aphasia, etc. This type of disease brings heavy economic pressure to the patient’s family and the entire society. The most common cause of stroke is cerebrovascular disease, of which hypertensive arteriosclerosis and atherosclerosis lead to vascular disease. The overall prognosis of patients with cerebrovascular disease with mild consciousness disorder is relatively good, and the survival rate is higher; the prognosis of patients with moderate consciousness disorder is poor, and most of them will have different degrees of sequelae. Patients with severe disturbance of consciousness have the highest mortality rate and the worst prognosis. Surviving patients have serious sequelae, which seriously affect the quality of life of patients. Figure 1 shows the flow chart of emergency care for patients with severe cerebrovascular disease.

Patients with severe cerebrovascular disease often have varying degrees of consciousness disturbance. In clinical practice, consciousness refers to the degree of awakening of the brain, which is the ability of the cerebral nervous system to respond to various internal and external stimuli in a timely manner. The subject of Consciousness Disorder is divided into the decline in the degree of arousal and the changes in the content of consciousness. The content of consciousness refers to the high-level neural activities of the cerebral cortex, including the mental activities of human perception, thinking, memory, orientation, emotion, and will activity. Consciousness content is manifested as confusion or delirium. The brainstem ascending activation system plays an important role in the maintenance of human consciousness. It receives various sensory signals and transmits them to the non-specific nucleus of the thalamus, and then projects to the extensive cerebral cortex, making the central nervous system in a state of excitement. Once a patient develops a disturbance of consciousness, indicating a critical condition, the prognosis of the patient is often poor. In clinical work, being able to recognize various types of consciousness disorders in patients in a timely and accurate manner, and be able to fully evaluate the patient’s condition and make correct treatments can enable patients with severe cerebrovascular disease to be rescued in time, and even turn the crisis into peace.

### EEG Physiological Monitoring for Brain Function Assessment

The cerebral cortex cells have spontaneous electrophysiological activities. When the brain is damaged by various reasons, it will show abnormal discharges, including changes in frequency and amplitude. EEG technology is to record these brain electrical activities through brain electrodes, and through the processing of computer technology, these brain electrical activities are turned into readable graphics that can more accurately reflect brain electrical activities. The potential on the EEG reflects the postsynaptic potential of the gray matter vertebral body cells, and the rhythm of the EEG is maintained by the brainstem network ascending activation system.

Electroencephalography technology uses sophisticated instruments to measure the rhythmic and spontaneous bioelectric activity of the cerebral cortex, and amplifies the recorded bioelectric signals by millions of times to understand the functional state of the brain. It is often used in the diagnosis and treatment of epilepsy in clinical practice. It is an important method for the diagnosis and classification of epilepsy. It can conveniently, objectively, safely, non-invasively and accurately evaluate the brain function of patients.

The placement of the EEG electrodes adopts the 10–20 system electrode placement method recommended by the International Electroencephalography Society. Its characteristic is that the arrangement of each brain electrode is proportional to the size and shape of the head, and the electrode name matches the brain anatomy zone. With the apex as the center of the circle, you draw straight lines to the temporal halves (divided into 10), and then make concentric circles with the halves of the sagittal line as the radius, and determine the electrode placement position according to the intersection point.

The condition of EEG is closely related to the metabolism of brain cells, and it is more sensitive to brain tissue damage caused by various causes. When brain cells undergo hypoxia and other pathological changes such as brain cell edema and necrosis, the EEG will show amplitude. The frequency changes, and the location of the lesion can be roughly located according to the position of the electrode, which plays an important role in the basic diagnosis process. For patients with severe cerebrovascular disease in clinic, an ordinary and short EEG cannot better reflect the patient’s brain function status. Long-term EEG can monitor the EEG physiological activities of severe patients for a long time at the bedside and provide long-term dynamic information.

### Clinical Analysis of Long-Term EEG

Severe cerebrovascular diseases are often caused by important responsible vascular lesions. In patients with common large-area cerebral infarctions, due to ischemia, edema, and necrosis of brain cells in the infarct focus, the brain cell potential in this part will change. Generally, the brain cells in the central area of the infarct focus have been necrotic, and the EEG generally shows a flat waveform with low amplitude. The brain cells adjacent to the peripheral edema zone of the lesion will also experience varying degrees of hypoxia. Generally, the EEG shows a low amplitude slow wave (θ wave or δ wave). Clinically, the severity of the patient’s condition can be judged based on the flat and slow wave range and amplitude.

**FIGURE 2:**
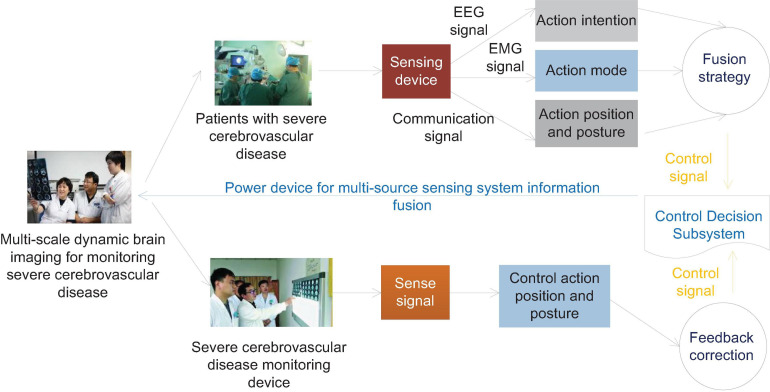
Schematic diagram of information fusion control of human body intention EEG and EMG multi-source sensing system.

In the early stage of the onset, some patients with cerebrovascular accidents, especially acute cerebral ischemic diseases, cannot find positive signs in time by neuroimaging examination alone. EEG is closely related to brain bio-metabolism and more accurately reflects the cerebral cortex. Brain bioelectrical activity can provide timely and accurate information on the state of brain functions closely related to abnormal brain metabolism. EEG is a more sensitive indicator for monitoring acute ischemic diseases. Infarction has important clinical diagnostic value and is worthy of promotion and application. When the local CBF is reduced to 20–30 ml/100 g/min, the EEG monitoring will appear abnormal. High amplitude waves can be seen to replace the background waves of the EEG. When the blood flow is reduced to 17 ml/(100 g min), synaptic activity will drop and the wave will disappear. When the blood flow is reduced to 10 ml/100 g/min, brain cells will be irreversibly damaged. Continuous EEG monitoring is of great significance for the early diagnosis of patients with acute vascular accidents. As patients with severe cerebrovascular diseases will have varying degrees of consciousness disturbances, physical examinations are poorly coordinated, and it is more subjective to judge the severity of the disease through neurological examinations, and EEG monitoring is extremely important. The human body intention perception based on brain muscle multi-source perception needs to judge the movement intention of brain electrical signals, so as to realize the active and passive coordinated control of brain muscle signals, as shown in Figure 2.

Abnormal EEG and brain topography show that the absolute power value of the fast-wave frequency band decreases, and the absolute power value of the slow-wave frequency band increases. Therefore, the EEG examination can more sensitively reflect the changes in brain function of patients before cerebral hemorrhage. The reason may be that brain damage has occurred in the early stage of hypertensive intracerebral hemorrhage. This examination provides reference and basis for early prediction and preventive treatment of the occurrence of intracerebral hemorrhage. Therefore, long-term EEG monitoring for patients with hypertension, especially the extremely high-risk group, is of clinical significance for the timely detection of cerebral hemorrhage.

The brain damage caused by non-convulsive epilepsy and NCSE and the damage of primary cerebrovascular disease are not only simply superimposed, but can make the condition further worse. After initial treatment of convulsive status epilepticus, if the interictal EEG shows epileptic discharges, periodic discharges, and persistent micro-seizure patterns, the risk of recurrence of convulsive status epilepticus increases by 5, 18, and 18, respectively. As we all know, electroencephalogram is an important auxiliary examination for the diagnosis of epilepsy, and it will not be described here. However, due to conditions and time constraints, the positive rate of diagnosis of epilepsy is not high. Some patients with severe encephalopathy have NCS in clinical practice. If there is no long-term EEG monitoring, it is difficult to find non-convulsive epilepsy in clinical work. Among the 90 patients in this study, there were 24 cases of epilepsy, and the incidence rate was 26.67%. However, only six cases had spastic seizures, the positive rate was only 6.6%, and the incidence of non-convulsive epilepsy was 20%, so non-convulsive epilepsy occurred in 20%. The incidence of convulsive epilepsy in patients with severe cerebrovascular disease is much higher than that of convulsive epilepsy. Long-term EEG is an important means to find non-convulsive epilepsy.

Long-term EEG monitoring can also be applied to the monitoring of epilepsy drug treatment. Insufficient dose of antiepileptic drugs cannot control epilepsy well. Excessive medication will increase the patient’s respiratory and cardiovascular diseases. Therefore, long-term EEG monitoring is extremely important.

### Research Objects for Surveillance of Severe Cerebrovascular Disease

The cases were selected from 90 patients with severe cerebrovascular disease in the Department of Neurology and Critical Care Medicine of the affiliated hospital of a medical university, including 42 male patients and 48 female patients, including 34 patients with cerebral hemorrhage and 56 patients with cerebral infarction. The exclusion criteria are shown in Table 1.

**TABLE 1:** Exclusion criteria.

Exclusion criteria number	Exclusion criteria description
1	Does not meet the basic standards
2	The patient’s family members ask to give up treatment after admission
3	Rectal body temperature < 32°C
4	Use drugs that significantly affect the determination of brain electrical activity before the experiment
5	Combined with serious diseases or factors that affect the judgment of brain function (such as hepatic encephalopathy, pulmonary encephalopathy, renal encephalopathy, toxic encephalopathy, autoimmune diseases, infectious diseases, brain tumors, etc.)
6	Nervous system examination is highly suspected of cerebral infarction but not confirmed by brain MRI
7	The patient does not breathe spontaneously and requires a ventilator to assist breathing
8	The patient’s limb jitter affects EEG acquisition
9	Past history of mental disorders

Telephone follow-up was uniformly used for 90 days. Among them, the patients who died within 90 days completed the follow-up, and the rest continued to follow-up until the patient was discharged 3 months later to understand the prognosis. Statistics of general conditions include age, gender, hypertension, blood lipids, type of cerebrovascular disease, GCS (Glasgow Coma Scale) score, EEG classification and typical EEG, etc., to analyze their relationship with the patient’s prognosis.

Patients with large-area cerebral infarction are limited to hospital emergency diagnosis by brain imaging examination as internal carotid artery or middle cerebral artery main trunk occlusion and unclassified cerebral vascular infarction. The cerebral infarction area exceeds a single lobe and the lesion area is more than 5 cm.

Intracerebral hemorrhage patients were admitted to the hospital by CT craniocerebral hemorrhage (mainly near the basal ganglia).

Based on brain imaging data, the computer calculates the area of the infarct lesions at each level (in cm^2^), multiplying the thickness of the septal layer of the image data (in cm) to calculate the volume of the infarct lesion. The brain blood volume of the patient is calculated according to the head MRI or CT, according to the maximum hematoma volume formula proposed by Tada’s.

### Research Methods for Monitoring Severe Cerebrovascular Disease

After admission, blood pressure was monitored repeatedly (measured by mercury sphygmomanometer). According to the diagnostic criteria for hypertension, patients with systolic blood pressure higher than 140 mmHg and diastolic blood pressure higher than 90 mmHg were defined as hypertensive patients.

According to the latest “Guidelines for the Prevention of Dyslipidemia in Adults in China,” each patient’s blood lipid level is divided: all blood samples were collected on an empty stomach, and the cholesterol (TC, TotalCholesterol) was 5.18–6.22 mmol/L for marginal increase, and cholesterol (TC) d is higher than 6.22 mmol/L; triglycerides (TG, TriGlycerides) higher than or equal to 2.26 mmol/L is higher. High density lipoprotein is (HDL, High Density Lipoprotein) < 1.04 mmol/L; low density lipoprotein is (LDL, Low Density Lipoprotein) ≥ 4.14 mmol/L.

All cases used the EEG monitoring system (SP3000) tendered from Shanghai Shijia Medical Equipment Co., Ltd. for continuous bedside EEG monitoring, and L-EEG (Low Electroencephalogram) tracing used FP1, FP2, C3, C4, T3, T4, O1, O2. The lead electrode is connected, and the lead is placed according to the international 10/20 system. The silver plate electrode is uniformly used. After the head is cleaned, the surface is degreased with an alcohol cotton ball, the electrode is glued with conductive paste, and then fixed with a medical mesh cap. We set the paper speed to 3 cm/s, the filter channel to 0.5–35 Hz, and record 12–24 h, and save for reading.

The included cases are in accordance with the Chinese guidelines for the prevention and treatment of cerebrovascular diseases, and try to maintain consistent treatment methods without affecting the efficacy. Patients with cerebral infarction are treated with conventional antiplatelet, improvement of cerebral circulation, lipid reduction, protection of nutrient brain cells, and dehydration; patients with intracerebral hemorrhage will undergo minimally invasive removal of intracerebral hematoma within the corresponding safe time window, followed by routine irrigation and drainage to the removal of needles.

Strictly follow the inclusion and exclusion criteria of the research objects, we communicate with the EEG physicians, and pay close attention to the EEG conditions of the patients during treatment to obtain comprehensive and reliable information. The discharged patients are followed up regularly every month according to the discharge time, and the recovery situation is investigated in detail according to the rehabilitation. Data collation and data processing are strictly proofread to prevent data errors from affecting the experimental results.

## Multi-Scale Dynamic Brain Imaging Monitoring Model for Severe Cerebrovascular Disease Based on Feature Fusion

### Densely Connected Backbone Network

Res Net and VGGNet (Visual Geometry Group Network) are currently popular backbone network types in the field of severe cerebrovascular disease monitoring, but the types of networks are difficult to meet our requirements for backbone network performance. Among them, due to the limitation of the number of network layers, VGGNet has relatively weak feature extraction capabilities and a large amount of network parameters; while the deep Res Net exhibits excellent feature extraction capabilities, it also has a large network scale and computational complexity. It is difficult to meet the requirements of real-time monitoring.

In the traditional convolutional neural network design philosophy, deepening the network depth is an effective method to improve the network feature extraction ability, but as the convolutional neural network deepens, the probability of information loss after passing through the multilayer network will also increase. The network is difficult to train. This is also the main reason for limiting the depth of traditional networks such as VGGNet. Res Net uses Skip Connection to establish a direct connection path from the low-level to the high-level of the network to improve this problem and increase the network depth. However, research has shown that in the process of training the residual network, if some layers are randomly discarded, the generalization ability of the network can be improved. This shows that the residual network structure has a certain degree of redundancy. Each layer in the network only learns few features, so fewer parameters can be used for learning in each layer. This also points out that a strict progressive hierarchical structure is not necessary for neural networks. Each layer in the network does not completely depend on the features of the immediately preceding layer, and it can also be learned from the features obtained by the previous layer. Dense Net (Densely Connected Convolutional Networks) is based on the above research.

Through its Dense Connectivity (Dense Connectivity) structure brings the characteristics of feature reuse, at the same depth, the number of network parameters and the amount of calculation are greatly reduced compared with other types of convolutional neural networks. The low-level information of the network using this structure can be effectively transmitted to the high-level, and the network depth can be deepened. Dense connections and residual connections have similarities, but there are also essential differences. The difference between the feature transfer of the residual network and the densely connected network will be explained below. Suppose there is an image y0 with an L-layer convolutional neural network. Each layer of the image must undergo a non-linear conversion operation Hl(.), Hl(.) is the representative function of the network operation set of this layer, where l refers to the current number of network layers, and the output of the first layer is defined as yl, then the feature transfer of the traditional feedforward convolutional network can be expressed by the following formula:


(1) $$\begin{array}{c} y_{l}=H_{l}\cdot \left(y_{l-1}\right) \end{array}$$

After adding the residual connection in Res Net, it can be expressed by the following formula:


(2) $$\begin{array}{c} y_{l}=H_{l}\cdot \left(y_{l-1}\right)+y_{l-1} \end{array}$$

In Dense Net, dense connections are used to further improve the transfer of information between layers. When the network joins the dense connection, the output of layer l can be expressed by the following formula:


(3) $$\begin{array}{c} y_{l}=H_{l}\cdot \left[y_{0},y_{1},\ldots ,y_{l-1}\right] \end{array}$$

Residual connection is to add the feature maps of the previous layer and the feature maps of the latter layer together, while dense connection is to splice the feature maps of the two together in a cascading manner, completely retaining the features output by the previous node.

**FIGURE 3:**
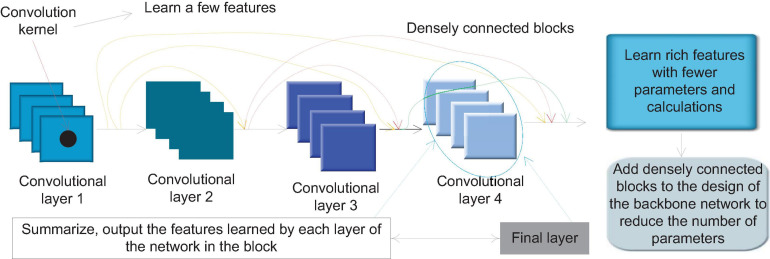
Schematic diagram of dense connection block structure.

The dense connection block is a special module in Dense Net. Figure 3 shows the internal structure of a typical densely connected block. In Dense Block, a basic structure is formed by a combination of several basic network operators such as convolution operations and activation functions. Each basic structure is fed forward through dense connections. The form of connection is connected to every other basic structure. This feature makes each convolutional layer in the densely connected block only need to use a small number of convolution kernels to learn a small number of features, and the final layer will summarize and output the features learned by each layer of the network in the block. This feature allows Dense Net to learn rich features with a small number of parameters and calculations. Therefore, we can add densely connected blocks to the design of the backbone network to achieve the need to reduce the number of parameters.

**FIGURE 4:**
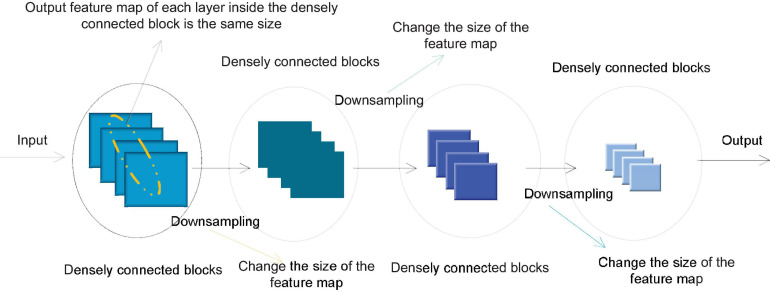
Densely connected network structure.

In order to avoid the inconsistency of the feature map size during splicing, a densely connected network usually consists of several densely connected blocks. The size of the feature map output by each layer within each densely connected block is the same. Downsampling is performed to change the size of the feature map. Figure 4 shows the structure of a typical densely connected network.

### Backbone Network Design

In view of the advantages of the densely connected network, the network has excellent feature extraction capabilities while the network parameters and calculations are small. This paper uses the design of dense connection blocks in the basic architecture of the algorithm backbone network, and builds a fast dense connection network F-Dense Net for the monitoring task of severe cerebrovascular disease in dynamic brain imaging in this paper. The main structure of F-Dense Net includes a simplified Stem block, four densely connected blocks and three conversion layers. In ordinary Dense Net, after the input layer, a convolutional layer with a convolution kernel size of 7 and a step size of 2 and a pooling kernel size of 3 and a maximum value pooling with a step size of 2 are connected. The structural Stem block can replace the initial two layers in the ordinary Desne Net, thereby reducing the loss of the original information of the input image. Compared with the structure of ordinary Dense Net, although the Stem block retains more original information, it will introduce a lot of extra calculations. Through further analysis, we combined a 7 × 7 cross-convolution with a step size of 2 in the traditional Desne Net and a 2 × 2 maximum pooling with a step size of 2 in the Stem block. This structure can calculate and reduce the loss of original information compared with the traditional structure.

We add a conversion layer between every two densely connected blocks instead of simply pooling down sampling. The conversion layer consists of a 1 × 1 convolutional layer and a 2 × 2 maximum pooling. Among them, the number of channels of the 1 × 1 convolutional layer is lower than the dimension of the feature map output by the previous densely connected block. This is to fuse the features of different channels while compressing network parameters. Maximum pooling is used to down-sample the feature map, and at the same time, it can better retain some local pattern features than average pooling.

In the traditional densely connected network, Re LU (Rectified Linear Unit) is used as the activation function. Re LU has many advantages over traditional activation functions such as Sigmoid function and Tanh function. However, the Re LU activation function itself has another problem, that is, the problem of dying Re LUs. In a network that uses Re LU as the activation function, suppose that some neural nodes learn a relatively large negative deviation term in the back propagation process, so that their input becomes less than or equal to 0, then Re LU will always be in a suppressed state. Their gradient is always 0, and the network cannot learn the features on these nodes. When there are many such nodes, many nodes in the network cannot learn effectively, and the actual feature extraction ability of the network is lower than the theoretical feature expression ability of the network. The Scaled Exponential Linear Units (SELU) activation function is an activation function with self-stabilizing characteristics. The formula of SELU is:


(4) $$\begin{array}{c} selu\left(y\right)=\lambda \cdot \left\{ \begin{array}{cc} y & y>0\\ a\cdot e^{y}-a & y\leq 0 \end{array}\right. \end{array}$$

The self-stabilization characteristic of SELU has an inhibitory effect on the gradient dispersion and gradient disappearance of the network. At the same time, the gradient of SELU is not 0 when the input is equal to 0, which avoids the node death problem of Re LU. In our research, we found that using SELU as the activation function in F-Dense Net has better performance than activation functions such as Re LU and Leaky Re LU, so we chose SELU as the activation function of F-Dense Net. In general, SELU has the following advantages: SELU suppresses the problems of gradient disappearance and gradient dispersion, making it very suitable for deep convolutional neural networks; SELU also avoids the dead zone problem of Re LU, and can give full play to the advantages of deep convolutional neural networks.

### Multi-Scale Network Structure

The feature image pyramid method improves the monitoring accuracy of severe cerebrovascular diseases at different scales by calculating features on each independent image scale. Therefore, this method introduces a lot of repetitive calculations in the training process, which brings a lot of extra time and computational resource overhead. If the feature image pyramid is used only during testing, it will lead to inconsistency between testing and training. The pyramid feature hierarchical structure method extracts features of different scales from different layers of the network for prediction, and does not bring additional calculations. However, the pyramid feature hierarchy method does not realize the fusion of multi-level features. The lack of high-level features on the feature map for small-scale severe cerebrovascular disease monitoring makes the semantic information insufficient and prone to misdetection of small-scale severe cerebrovascular disease.

This paper adopts a new multi-scale feature extraction method, Feature Pyramid Networks (FPN) as a method to achieve multi-scale feature extraction. The FPN structure only brings a small amount of additional computational overhead on the premise of multi-level feature fusion. We improve the network architecture of F-Dense Net through the FPN structure to obtain a convolutional neural network with multi-scale feature expression capabilities. The FPN structure mainly consists of three parts: bottom-up path, top-down path and horizontal connection. The network that joins the FPN structure can solve the problem of multi-level feature fusion and extraction through the combination of these three feature transmission paths. Through the horizontal connection, the network can merge the high-level feature map with less low-level feature information and more semantic information in the top-down path and the low-level feature map with more low-level feature information and less semantic information in the bottom-up path, and this structure also provides the network with the ability to predict on multiple scale feature maps.

**FIGURE 5:**
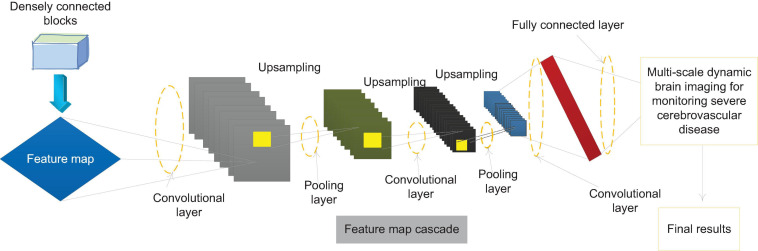
Implementation method of feature pyramid based on MF-Dense Net.

This paper introduces FPN on the basis of F-Dense Net to improve the network structure, and designs a network structure that supports three-scale prediction. The specific structure is shown in Figure 5. The bottom-up path is the forward propagation process of F-Dense Net, and the top-down path is through the last layer of F-Dense Net. The semantic information output is the richest. High-level feature maps are sampled and then followed by the bottom-up path mesoscale. The matched feature maps are combined by horizontal and horizontal connections. In the feature pyramid network, those layers whose output feature map size can be said to be at the same stage of the network, and each stage can output an independent feature map for monitoring. In F-Dense Net, the output feature map size of each densely connected block is the same, which means that all layers in a densely connected block can be regarded as the same stage in the feature pyramid. Therefore, we choose the output of the last layer of the second, third, and fourth densely connected blocks as the reference for the feature map. This is considering that the last layer of each densely connected block has the learned features of all layers in the block, and at the same time has the richest semantic information.

In the network designed in this paper, the nearest neighbor method is used for the up-sampling of the feature map, and the up-sampling multiple is 2. In addition, we have made some improvements to the FPN method. The traditional feature pyramid horizontal connection needs to adjust the number of channels of the two feature maps to the same, and then add pixels on each channel to generate the final feature map. In this paper, combining the characteristics of the densely connected network, different horizontal connection methods are adopted. The two feature maps are merged into a new one with the same size and the same dimension as the sum of the dimensions of the two original feature maps using the feature map cascade method in the dense connection. The feature map better retains the information of the two feature maps. Finally, a 1 × 1 convolutional layer is added after the new feature map. On the one hand, it is to alleviate the aliasing effect caused by upsampling. It can strengthen the fusion of different channel features, while compressing the number of parameters and reducing the amount of network calculations.

### Spatial Pyramid Pooling

Through research, we found that the multi-scale feature fusion in MF-Dense Net mainly focuses on the fusion between the global features of different levels of convolutional layers, and there is no effective fusion and extraction of multi-scale local features on the same convolutional layer. In response to this problem, we propose an improved spatial pyramid pooling structure to improve this problem.

The spatial pyramid pooling structure can generate fixed-size outputs from all sizes of inputs. Spatial pyramid pooling is generally connected between the last convolutional layer of the convolutional neural network and the fully connected layer to meet the requirements of the fully connected layer for the dimensionality of the input data. The convolutional layer produces an output of w^∗^h^∗^d, where w^∗^h is the size of the feature map, and d is the dimension, which is determined by the number of filters in the convolutional layer. After inputting this feature map into the spatial pyramid pooling structure, after pooling with different core sizes and step lengths, three feature maps of 4^∗^4^∗^d, 2^∗^2^∗^d, and 1^∗^1^∗^d are obtained, and then the feature map is converted into a 1-dimensional vector and stitched together, then no matter what the size of the input feature map is, the output of the same size will be generated after the spatial pyramid pooling, that is, 1 × 1 × 21d.

The spatial pooling pyramid merges the features pooled by different pooling core sizes during the operation process, and realizes the fusion of multi-scale local area features on the same layer of feature maps. However, the traditional spatial pyramid converts multiple feature map information into one-dimensional vectors and outputs them to the fully connected layer, which is not applicable in our method. Therefore, a spatial pyramid method of outputting three-dimensional feature maps is used in this article.

In the improved spatial pyramid pooling of feature maps, the input spatial pyramid pooling feature maps are pooled with multiple different pooling core sizes. In ordinary spatial pyramid pooling, the pooling step size is generally greater than 1, which plays the role of downsampling the feature map; and in this improved method, the step size of the three pooling layers used is all 1. The pooling core size can be set to 1, 1/2, and 1/3 times the edge length of the feature map (rounded up for non-integer cases). The pooling form uses maximum value pooling, and after pooling, a new feature map with the same size is the input feature map (w × h × d), and then cascades these three feature maps together in the dimension direction to obtain a w × h × 3d feature map, which is obtained by cascading fusion of multi-scale local area features.

In terms of specific implementation, we added a spatial pooling pyramid module after the last densely connected block of MF-Desne Net. We first use 1 × 1 convolution to reduce the dimensionality of the feature map output by the last layer of MF-Desne Net, and then input it into the improved spatial pyramid pooling structure to obtain a new feature map that incorporates multi-scale local features. Subsequently, the real parameter compression and multi-channel feature fusion of the new feature map are performed by 1 × 1 convolution. Finally, the feature map is cascaded with the original feature map output by MF-Dense Net, and the 1 × 1 convolution is used to reduce the dimension to 512 dimensions and input into the monitoring sub-network. By adding a spatial pooling pyramid structure, the improved backbone network can not only achieve multi-level feature fusion, but also multi-scale local feature fusion in the same dimension, and improve the accuracy of object monitoring through richer feature information.

### Enhanced Utilization of Low-Level Features

Through the observation of the structure of MF-Dense Net, we found that the feature map output by the first densely connected block is not fully utilized in the feature extraction process. Because dynamic brain images often contain a large number of small-scale severe cerebrovascular diseases, the information of these severe cerebrovascular diseases is likely to be lost after multi-layer convolution operations, which makes it difficult to accurately monitor; compared to high-level feature maps, the feature map output by a densely connected block contains more low-level features and detailed information, which retains more feature patterns that can reflect small-scale severe cerebrovascular diseases and location information of some severe cerebrovascular diseases. Therefore, strengthening the use of low-level features can improve the monitoring effect of small-scale severe cerebrovascular diseases, and at the same time improve the positioning accuracy of some severe cerebrovascular diseases to be monitored. In order to make full use of the information of the feature map, we tried from different directions. The method of adding a new feature map to the top-down path increases the amount of calculation greatly and has a great impact on the monitoring speed; while the third feature map continues to be up-sampled. The method of feature fusion will also bring more extra calculations, and the aliasing effect caused by continuous up-sampling of the feature map will affect the effectiveness of the information on the high-level feature map to a certain extent. Therefore, we choose to merge the feature maps output by the first densely connected block through horizontal connection without up-sampling the third feature map.

Due to the different sizes of the two feature maps, the area of the feature map output by the first densely connected block is four times that of the third feature map in the top-down path, and the horizontal connection needs to be on several feature maps of the same size. Therefore, it is necessary to convert the larger feature map to the size of the smaller feature map to merge the two feature maps. Generally speaking, downsampling methods such as pooling can be used to reduce the size of the feature map, but this will cause a lot of loss of low-level detail information. In order to better preserve the low-level information of the feature map output by the first densely connected block, we use the Reorg Layer to reconstruct the feature map to change the feature map size, and then perform the fusion of the feature map through horizontal connections.

**FIGURE 6:**
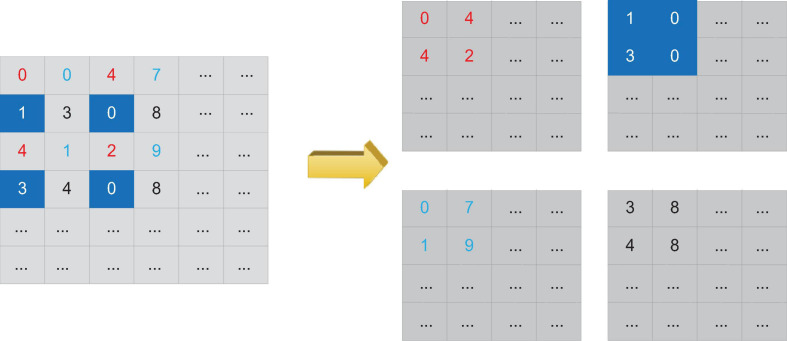
Schematic diagram of reconstruction operation.

The reconstruction operation can change the size of the feature map without losing data. The reconstruction process is shown in Figure 6. Assuming that the reconstruction layer obtains a feature map input of 2w^∗^2h^∗^d, if the step size of the reconstruction operation is set to 2, then the reconstruction layer can convert the input feature map into a feature map output of size w^∗^h^∗^4d. Compared with using the pooling layer to down-sample the feature map, changing the size of the feature map through the reconstruction layer retains more low-level features, which is more conducive to the monitoring of small-scale severe cerebrovascular diseases. At the same time, these low-level features also contain more location information.

## Analysis of the Results of Monitoring and Prognosis of Severe Cerebrovascular Disease

### The Relationship Between APACHEII Score and Overall Severe Cerebrovascular Disease

**FIGURE 7:**
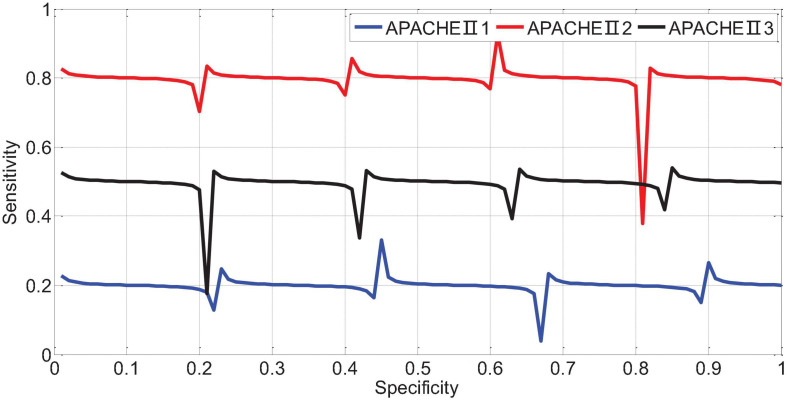
ROC curve of APACHEII1, APACHEII2, and APACHEII3 predicting the risk of death.

APACHEII1, APACHEII2, and APACHEII3 in the death group were higher than those in the survival group, and the difference between the two was statistically significant (*p* < 0.01). The change rate of APACHEII in the survival group was higher than that in the death group, and the difference between the two was statistically significant (*p* < 0.01). The area under the curve (AUR) of the ROC (Receiver Operating Curve) of APACHEII1 is 0.22; the AUR of the ROC curve of APACHEII2 is 0.83; the AUR of the ROC curve of APACHEII3 is 0.54, as shown in Figure 7. The accuracy of APACHEII1, APACHEII2, and APACHEII3 on the overall prognosis of patients is better, and APACHEII2 has the best accuracy.

The APACHEII1, APACHEII2, and APACHEII3 of the overall patients were divided into cut-off values of 19, 19, and 17, respectively. The score ≥ the cut-off value is the high-risk group, and the score < cut-off value is the low-risk group. The fatality rate of the high-risk group is significantly higher than that in the low-risk group, the difference between the two groups was statistically significant.

**FIGURE 8:**
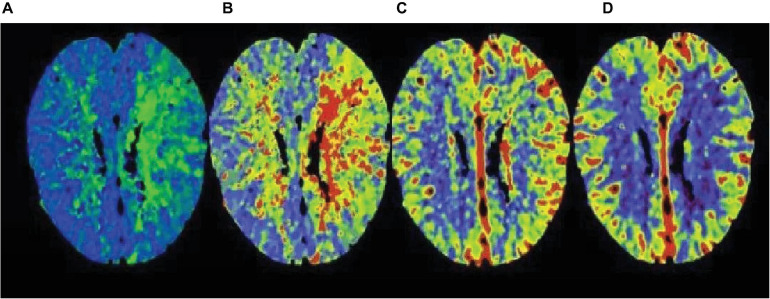
Image performance of severe cerebrovascular disease based on multi-scale dynamic brain imaging. **(A)** Imaging manifestation of severe cerebrovascular disease in 18 h brain imaging; **(B)** Imaging manifestation of severe cerebrovascular disease in 36 h brain imaging; **(C)** Imaging manifestation of severe cerebrovascular disease in 54 h brain imaging; **(D)** Imaging manifestation of severe cerebrovascular disease in 72 h brain imaging.

The APACHE II that showed an upward trend within 72 h was divided into high-risk group, and those with a downward trend were divided into low-risk group. The fatality rate of the high-risk group was 67.86%, the fatality rate of the low-risk group was 17.70%, and the fatality rate of the high-risk group was significantly higher than that of the low-risk group. The difference between the two groups was statistically significant. The imaging manifestations of severe cerebrovascular diseases based on multi-scale dynamic brain imaging are shown in Figure 8.

**FIGURE 9:**
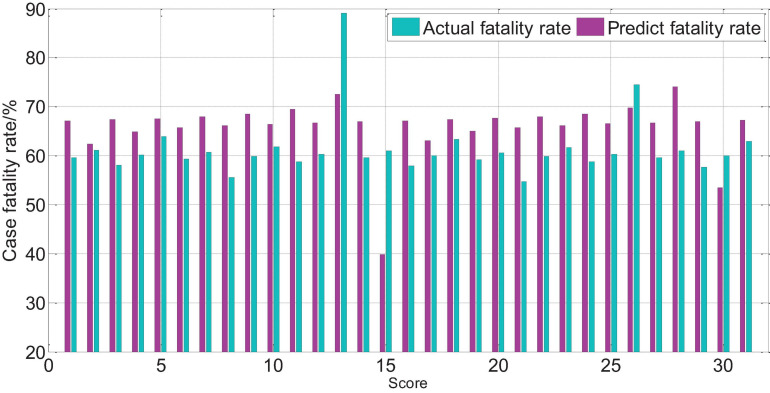
APAHCEII1 score predicts overall mortality and actual mortality.

**FIGURE 10:**
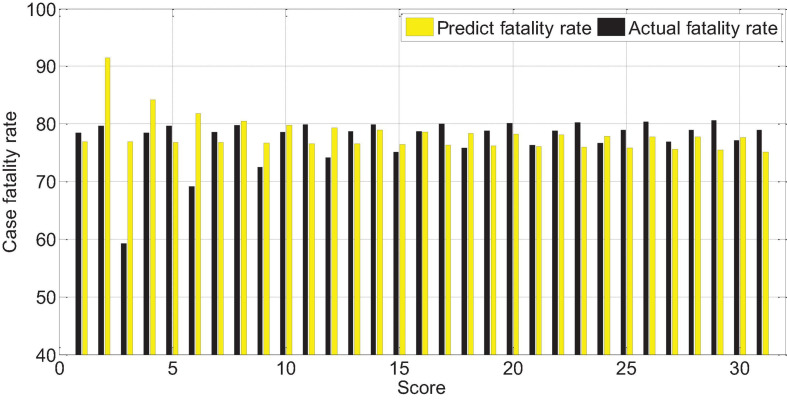
APAHCEII2 score predicts overall mortality and actual mortality.

**FIGURE 11:**
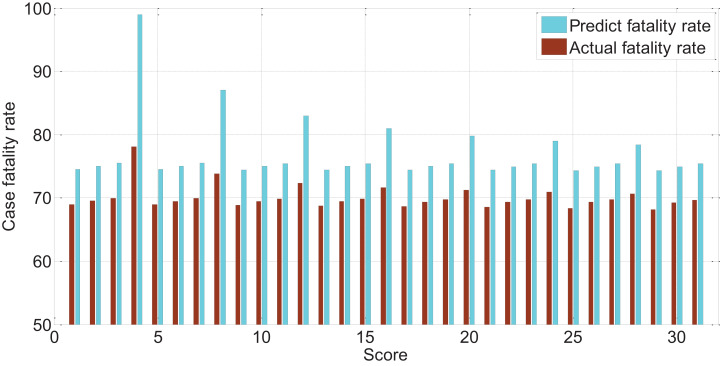
The relationship between APAHCEII3 score predicting overall mortality rate and actual mortality rate.

The APACHEII1 score is correlated with the patient’s prognosis (*r* = −0.47, *p* < 0.01). The predictive value of the APACHEII1 score for the overall mortality of severe cerebrovascular disease is generally higher than the actual mortality, as shown in Figure 9. APACHEII2 is correlated with prognosis (*r* = −0.63, *p* < 0.01), as shown in Figure 10. APACHEII3 is correlated with prognosis (*r* = −0.54, *p* < 0.01). The higher the score, the higher the mortality rate. The predicted value of APACHEII3 for the overall mortality rate is higher than the actual mortality rate, as shown in Figure 11.

### The Relationship Between APACHEII Score and Severe Cerebral Hemorrhage

The APACHEII1, APACHEII2, and APACHEII3 scores of patients in the death group were higher than those in the survival group, and the difference between the two was statistically significant (*p* < 0.01). The change rate of APACHEII in the survival group was higher than that in the death group, and the difference between the two was statistically significant (*p* < 0.01).

**FIGURE 12:**
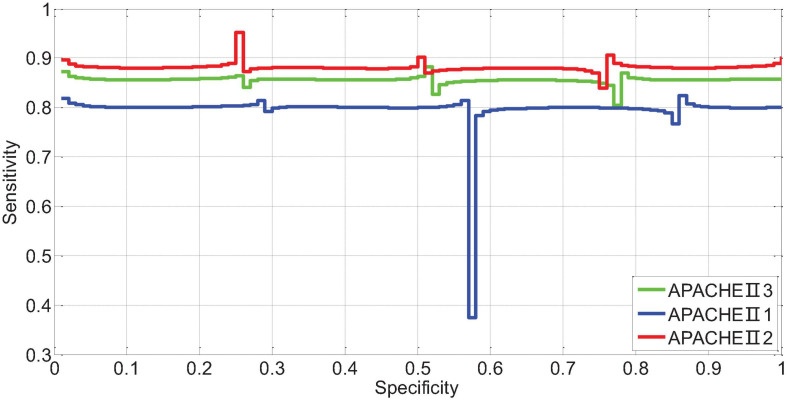
The ROC curve of APACHEII1, APACHEII2, APACHEII3 predicting the risk of death from severe cerebral hemorrhage.

The AUR of the ROC curve of APACHEII1 is 0.81; the AUR of the ROC curve of APACHEII2 is 0.88; the AUR of the ROC curve of APACHEII3 is 0.85. This shows that APACHEII1, APACHEII2, and APACHEII3 have good prognostic accuracy, and APACHEII2 has the best accuracy, as shown in Figure 12.

The scores corresponding to the Youden index of APACHEII1, APACHEII2, and APACHEII3 are 18, 19, and 18 points, respectively, indicating that for patients with severe cerebral hemorrhage, the first 24 h after admission to the NICU are higher than 18 points, and the internal score was higher than 19 points, and the score did not drop below 18 points in the third 24 h, and the mortality rate was high.

The APACHEII1, APACHEII2, and APACHEII3 of the overall patients were divided into cut-off values of 18, 19, and 18, respectively. The score ≥ the cut-off value is the high-risk group, and the score < cut-off value is the low-risk group, and the fatality rate of the high-risk group is significantly higher. In the low-risk group, the difference between the two groups was statistically significant. The APACHE II that showed an upward trend within 72 h was divided into a high-risk group, and those with a downward trend were divided into a low-risk group. The fatality rate of the high-risk group was significantly higher than that of the low-risk group, and the difference between the two groups was statistically significant.

**FIGURE 13:**
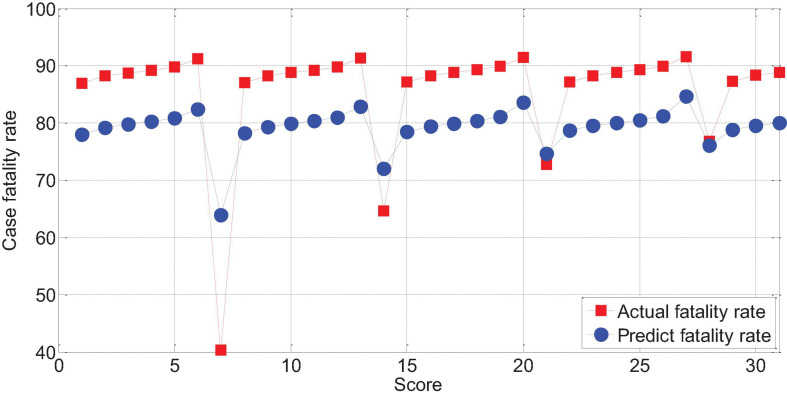
The relationship between APACHEII1 and the mortality of patients with severe cerebral hemorrhage.

The score of APACHEII1 is correlated with the prognosis of patients (*r* = -0.65, *p* < 0.01). The higher the score, the higher the risk of death. Overall, the predictive value of the APACHEII1 score for the fatality rate of severe cerebral hemorrhage is lower than the actual value of the fatality rate, as shown in Figure 13.

**FIGURE 14:**
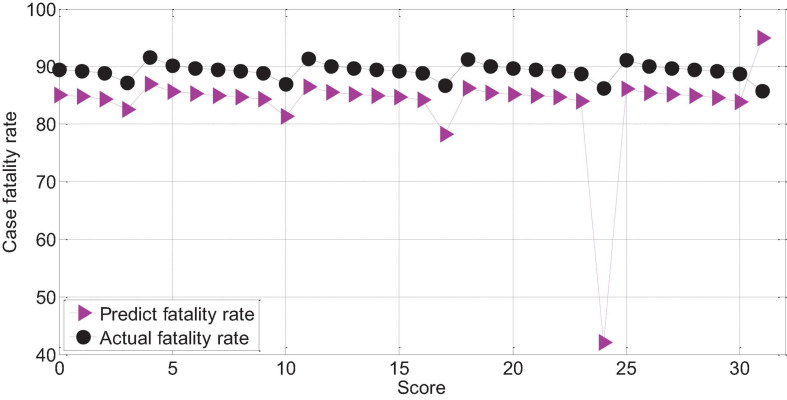
APAHCEII2 predicts the relationship between mortality and actual mortality in patients with severe cerebral hemorrhage.

The APACHEII2 score is correlated with the patient’s prognosis (*r* = -0.78, *p* < 0.01). The higher the APACHEII2 score, the higher the risk of death. The predicted value of the fatality rate for severe cerebral hemorrhage with APACHEII2 score below 30 points is always lower than the actual fatality rate. The actual mortality rate is lower than the predicted mortality rate above 30 points, as shown in Figure 14.

**FIGURE 15:**
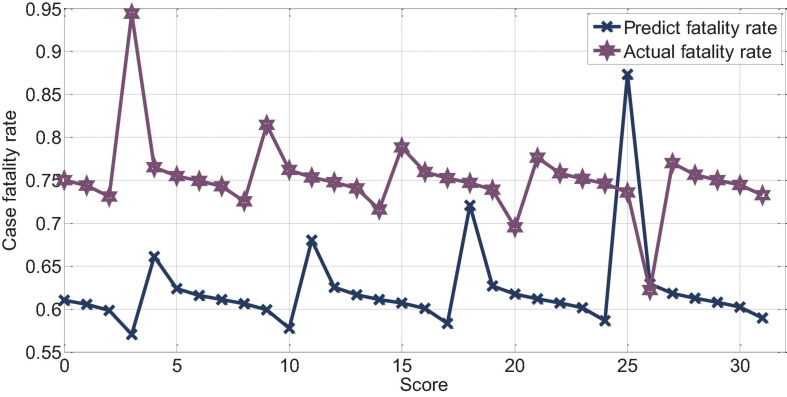
APAHCEII3 predicts the relationship between mortality and actual mortality in patients with cerebral hemorrhage.

The score of APACHEII3 was correlated with the prognosis of patients (*r* = −0.72, *p* < 0.01). The APACHEII3 score is less than 24 points, and the predicted value of the fatality rate of severe cerebral hemorrhage is always lower than the actual fatality rate. The actual mortality rate is lower than the predicted mortality rate at 24–26 points, as shown in Figure 15.

### Logistic Regression Analysis and Comparison of Evaluation Methods and Prognosis

**FIGURE 16:**
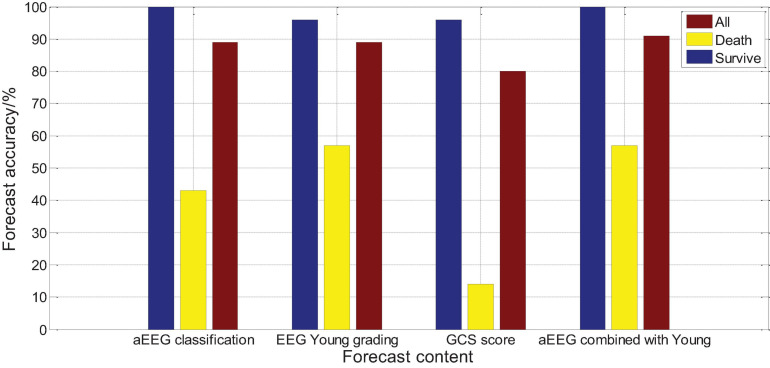
Comparison of evaluation methods and prognostic logistic regression analysis.

Logistic regression analysis was performed on the aEEG (Ambulatory Electroencephalogram) classification and prognosis, and the χ^2^ test was performed on the fit of the regression equation model, *P* = 0.004, indicating that the model was well fit and the regression equation was significant. Logistic regression analysis showed that a EEG classification had an accuracy rate of 100% for survival prognosis, an accuracy rate of 42.5% for death prognosis, and an accuracy rate of 87.1% for comprehensive prognosis. Logistic regression analysis was performed on the Young grading and prognosis of EEG, and the χ^2^ test was performed on the fit of the regression equation model, *P* = 0.003, indicating that the fit of the model was good and the regression equation was significant. Logistic regression analysis showed that the accuracy of EEG Young classification for survival prognosis judgment was 97.7%, the accuracy of death prognosis judgment was 58.9%, and the accuracy of comprehensive prognosis judgment was 89.1%. Logistic regression analysis was performed on the GCS score and prognosis, and the fit of the regression equation model was tested by χ^2^, *P* = 0.016, indicating that the model fit is acceptable and the regression equation is meaningful. Logistic regression analysis showed that the accuracy of GCS score for survival prognosis judgment was 97.2%, the accuracy of death prognosis judgment was 13.9%, and the accuracy of comprehensive prognosis judgment was 79%, as shown in Figure 16. The results show that aEEG classification and EEG Young classification are more accurate in prognostic judgment than GCS score; and aEEG classification combined with EEG Young classification can improve the prediction accuracy.

## Conclusion

Severe cerebrovascular disease has an extremely high disability rate and mortality rate, and correct assessment of its prognosis is of great help to adopt correct treatments and reduce the disability rate and mortality rate. In today’s era of highly developed imaging, the majority of patients with cerebrovascular disease are the first choice for cranial computed tomography (CT) or magnetic resonance imaging (MRI) to assess the patient’s prognosis through objective images. This article studies the correlation between long-term EEG classification and typical EEG configuration and patient prognosis, in order to find a more objective clinical method for evaluating the condition and prognosis of patients with severe cerebrovascular disease. In this paper, a study on the feature extraction of critical cerebrovascular disease monitoring tasks based on multi-scale dynamic brain imaging is carried out. In view of the large-scale changes and diverse characteristics of severe cerebrovascular diseases in dynamic brain images, the idea of improving the monitoring effect through multi-scale fusion of the characteristics of severe cerebrovascular diseases is proposed. We designed a fast and robust dense connection network F-Desne Net with excellent feature extraction capabilities, and combined with the feature gold tower network architecture, we built a severe cerebrovascular disease that can extract multi-layer fusion features and output multi-scale feature maps. Based on MF-Desne Net, a fast and accurate end-to-end dynamic brain imaging monitoring algorithm for severe cerebrovascular disease MDRD is designed. In addition, we designed an improved method on the basis of MDRD. Improved feature pyramid pooling is added to improve the network’s multi-scale local feature fusion ability; the use of low-level features of the network is enhanced to improve the network’s ability to extract basic feature patterns and detailed information of the image. The improved method can effectively improve the monitoring performance of the MDRD method. This study found that APACHEII1, APACHEII2, and APACHEII3 in the death group were higher than those in the survival group. The change rate of APACHEII in the survival group was higher than that in the death group (*p* < 0.01). APACHEII in different periods was correlated with prognosis. APACHEII has a good predictive value for the prognosis of severe cerebrovascular disease, and has clinical practice value for predicting the risk of death. Dynamic observation of the changes in APACHEII score is of significance for the prediction and prognostic evaluation of severe cerebrovascular disease in neurology.

## Data Availability Statement

Publicly available datasets were analyzed in this study. This data can be found here: https://brainweb.bic.mni.mcgill.ca/.

## Ethics Statement

The studies involving human participants were reviewed and approved by the Hanyang Hospital, Wuhan University of Science and Technology. The patients/participants provided their written informed consent to participate in this study.

## Author Contributions

All authors listed have made a substantial, direct and intellectual contribution to the work, and approved it for publication.

## Conflict of Interest

The authors declare that the research was conducted in the absence of any commercial or financial relationships that could be construed as a potential conflict of interest.
